# Pre-Clinical Drug Testing in 2D and 3D Human In Vitro Models of Glioblastoma Incorporating Non-Neoplastic Astrocytes: Tunneling Nano Tubules and Mitochondrial Transfer Modulates Cell Behavior and Therapeutic Respons

**DOI:** 10.3390/ijms20236017

**Published:** 2019-11-29

**Authors:** Prospero Civita, Diana M. Leite, Geoffrey J. Pilkington

**Affiliations:** 1 Brain Tumour Research Centre, Institute of Biological and Biomedical Sciences (IBBS), School of Pharmacy and Biomedical Sciences, University of Portsmouth, White Swan Road, Portsmouth PO1 2DT, UK; d.leite@ucl.ac.uk; 2 Department of Chemistry, University College London, 20 Gordon Street, Christopher Ingold Building, London WC1H 0AJ, UK

**Keywords:** glioblastoma, nano tunneling, in vitro 3D model, human serum supplementation, astrocytes, hyaluronic acid, extracellular matrix, tumour microenvironment, drug screening

## Abstract

The role of astrocytes in the glioblastoma (GBM) microenvironment is poorly understood; particularly with regard to cell invasion and drug resistance. To assess this role of astrocytes in GBMs we established an all human 2D co-culture model and a 3D hyaluronic acid-gelatin based hydrogel model (HyStem™-HP) with different ratios of GBM cells to astrocytes. A contact co-culture of fluorescently labelled GBM cells and astrocytes showed that the latter promotes tumour growth and migration of GBM cells. Notably, the presence of non-neoplastic astrocytes in direct contact, even in low amounts in co-culture, elicited drug resistance in GBM. Recent studies showed that non-neoplastic cells can transfer mitochondria along tunneling nanotubes (TNT) and rescue damaged target cancer cells. In these studies, we explored TNT formation and mitochondrial transfer using 2D and 3D in vitro co-culture models of GBM and astrocytes. TNT formation occurs in glial fibrillary acidic protein (GFAP) positive “reactive” astrocytes after 48 h co-culture and the increase of TNT formations was greater in 3D hyaluronic acid-gelatin based hydrogel models. This study shows that human astrocytes in the tumour microenvironment, both in 2D and 3D in vitro co-culture models, could form TNT connections with GBM cells. We postulate that the association on TNT delivery non-neoplastic mitochondria via a TNT connection may be related to GBM drug response as well as proliferation and migration.

## 1. Introduction

Glioblastoma (GBM) is the most common and most aggressive form of primary brain tumour in adults, carrying a dismal prognosis. Current standard of care for newly diagnosed GBM remains complex consisting of maximal surgical resection, localized radiotherapy followed by adjuvant temozolomide (TMZ) [1]. In the last decade, cellular heterogeneity of GBM has been extensively studied, as evidenced by the numerous single-cell RNA-seq publications describing the intra-tumoral and inter-tumoral variability of cell types and histological compartments in GBM [2,3,4,5].

The GBM tumour microenvironment (TME) is characterized by complex networks of cancer cells and stromal cells such as astrocytes, microglia, and endothelial cells and an extensive release of soluble factors [6], which interact with the tumour and generate permissive conditions for tumour growth [7]. Recent studies [8,9,10,11,12,13,14] have explored the field of cell-to-cell interaction and the potential exchange of cellular content between non-neoplastic and tumour cells but only limited evidence has been described in GBM [15,16]. 

Astrocytes, the most numerous non-neuronal cells in the central nervous system (CNS), comprise approximately 50% of the human brain volume [17]. They dynamically respond to changes in the brain environment. While altered astrocytic function is well recognized as a primary contributory factor to a number of neurological diseases, their physiological function is to maintain homeostasis of the brain microenvironment [17,18]. For example, astrocytic cells are directly implicated in the structure/organization of the brain-blood barrier (BBB) and modulating cerebral blood flow [18]. Astrocytes also play important roles in neuronal function coordinating synapse formation during development [19], they also protect neurons by removing metabolic waste products [20], and detect and integrate signals of neuronal damage and inflammation to regulate the neuro inflammatory response [21]. In addition to the physiological roles that astrocytes play in the healthy CNS, during pathological conditions, such as trauma, ischemia, or stroke, astrocytes become activated, upregulating glial fibrillary acidic protein (GFAP) expression and showing a characteristic polarization with the formation of long processes [22].

In GBM, astrocytes directly interact with tumour cells and communicate via gap junctions, leading to increased intracellular calcium and resistance to treatments [23]. Indirect or paracrine communication with surrounding cells often occurs via secretion of exosomes known to carry miRNAs to target key tumour suppressor genes in tumour cells and surrounding microenvironment [24]. Interestingly, Lin J et al. identified subpopulations of astrocytes in the adult brain and in gliomas that respectively contribute to synaptogenesis and tumour pathophysiology, providing a blueprint for understanding diverse astrocyte contributions to neurological disease [25]. Recently the chemo protective role of astrocytes has been investigated in a co-culture with human breast cancer cells, lung cancer cells, or melanoma cells [26,27], establishing the role of reactive astrocytes in brain metastases [28]. Although several publications have investigated the mechanism of chemo resistance of GBM proposing different hypotheses, e.g., communication by growth factor release and paracrine signals, extracellular vehicles (EVs), membrane protrusions, and gap junctions (GJs) [29], the mechanisms by which astrocytes promote tumour progression and drug resistance require further investigation.

The latest discoveries in cancer biology have indicated new mechanisms for cell-to-cell communications, namely tunneling nanotubes (TNTs). TNT structure was initially characterized in Rustom’s lab in 2004 as long, thin, filamentous membrane extensions acting as delivery tubes between two cells [30]. Subsequently, these have been physically described in numerous cell types [31,32,33,34], including rat astrocytes and C6 glioma cells [9], PC12 cells [35], and murine neuronal cells [36]. Formation of TNTs has been demonstrated, both in vivo and in vitro, to facilitate direct intercellular communication between malignant and normal cells [33] suggesting that TNTs may promote cancer cell resistance, recurrence and metastasis [33,37]. However, the mechanisms of de novo formation of TNT and maintenance in refractory cancer are still unexplored. In addition, whether there exist truly cancer-specific components of TNTs remains to be determined in order to provide selective targets for cancer therapy. 

Previous studies have shown that preferential mitochondrial transfer through TNTs from stromal cells into cancer cells modulates chemo resistance [37,38]. With regard to astrocytes, despite being the most abundant glial cells in the brain, and therefore highly abundant within the GBM microenvironment, their impact on the tumour microenvironment is yet to be fully understood.

Based on our considerable experience in developing 3D human cell in vitro models in the context of GBM invasion [39], BBB-mediated drug delivery [40], brain metastasis [41,42], and drug screening in relevant in vitro all human models [43], we established 2D and 3D in vitro models in a hyaluronic acid-gelatin based hydrogel mimicking TME to investigate the impact of astrocytes within the GBM microenvironment. By exploring the significance of astrocytes in the TME, we demonstrated that astrocytes physically interact with GBM cells reducing drug sensitivity to TMZ, vincristine (VCR), and clomipramine (CLM) by developing TNT connections. We also found that trafficking of mitochondria through TNTs between astrocytes and GBM cells provides insights into the mechanism underlying the capacity of astrocytes to promote GBM aggressiveness and resistance to cytotoxics in 2D and 3D in vitro co-culture models, and that this mitochondria transfer could be implicated in apoptotic rescue in GBM cells.

## 2. Results

### 2.1. Cell Trace Dyes Label GBM and Astrocytes Tracing Cell Proliferation

**Figure 1:**
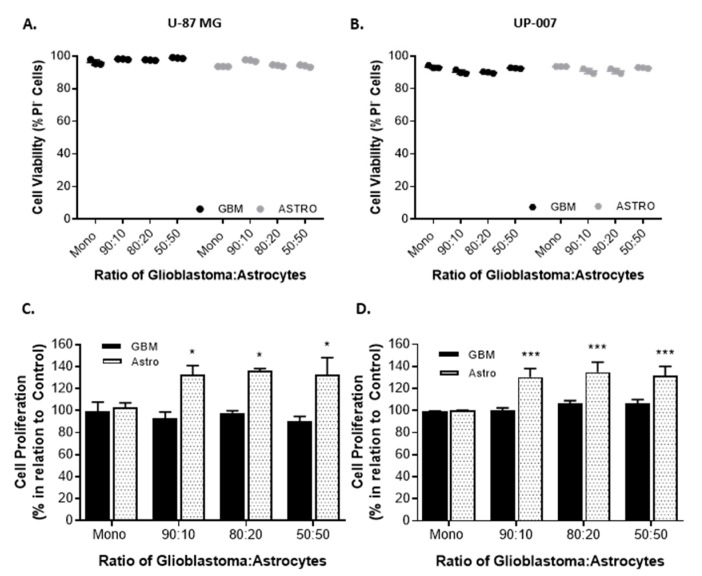
Viability and proliferation of glioblastoma (GBM) and astrocytes (Astro) growing in 2D contact co-culture. (**A**,**B**) Cell viability expressed as the percentage of propidium iodide (PI) negative cells. U-87 MG (**A**) and UP-007 (**B**) cells cultured in a contact co-culture with astrocytes at a GBM to astrocyte ratio of 90:10, 80:20, and 50:50. (**C**,**D**) Proliferation of GBM and astrocytes in a contact co-culture at ratios of 90:10, 80:20, and 50:50. U-87 MG (**C**) and UP-007 (**D**) proliferation expressed in relation to the cells growing in a monoculture in 2D. Mean ± SEM (*n* = 3). * *p* < 0.05, *** *p* < 0.001 compared to monoculture of either GBM or astrocyte.

In order to establish co-culture models to mimic the TME and investigate the influence of non-neoplastic astrocytes on the behavior of GBM cells, we optimized cell labelling using Cell Trace dyes in order to distinguish both cell populations as per previous publication [43]. Two GBM cell lines (U-87 MG, UP-007) were stained using Cell Trace carboxyfluorescein succinimidyl ester (CFSE), (Figure 1A,B; Figure S1) and the percentage of CFSE-positive cells remained >95% during 7 days of culture demonstrating low toxicity even using a high concentration of dyes (10 µM). Along with GBM cells labelled with Cell Trace CFSE, the human astrocyte cell line (UP-010) was labelled with Cell Trace Far Red to obtain a reliable labelling that allowed us to track both GBM cells and astrocytes. Using Cell Trace Far Red (1 μM), astrocytic cells retained Far Red-positive during 9 days in culture and remained viable when compared to unstained cells (Figure S2).

### 2.2. Astrocytes Influence GBM Proliferation and Invasion

**Figure 2:**
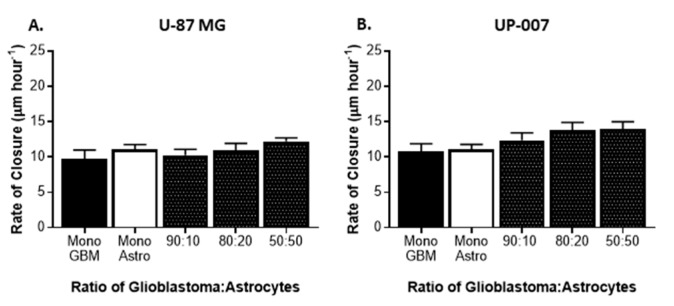
Motility of glioblastoma (GBM) in 2D co-culture with astrocytes. Scratch assay and rate of closure with U-87 MG (**A**) and UP-007 (**B**) co-culture with astrocytes at a GBM to UP-010 ratio of 90:10, 80:20, and 50:50. Mean ± SEM (*n* = 3).

To directly evaluate the effect of astrocytes on GBM behavior, we established co-cultures of CFSE-positive GBM cells and Far Red-positive astrocytes at ratios of 90:10, 80:20, and 50:50. The purpose of the use of different GBM to astrocyte ratios was based on the evidence that astrocytes comprise approximately 50% of the cells in the brain [17,44]. As a control, GBM and astrocytes were grown in a monolayer. In all the co-culture ratios of GBM to astrocytes used, both cells revealed the ability to grow together establishing direct contact and, as displayed in Figure 1A,B both cells remained viable (viability >95%) in co-culture. To study the effects of co-culture of astrocytes on GBM proliferation, the fluorescence intensity of each Cell Trace (CFSE or Far Red) was used as an indirect measure of proliferation following flow cytometry analysis (Figure 1C,D). In the two GBM cell lines, the presence of astrocytes in culture resulted in a slight increase in GBM cell proliferation, in UP-007 while in the U87-MG the presence of astrocytes did not have any effect on GBM proliferation. Interestingly, an increase in the proliferation of UP-010 when in co-culture with GBM was found (Figure 1C,D). The effect of astrocytes on GBM motility was also assessed using a wound-healing scratch assay (Figure 2). Initially, we performed a scratch-wound assay on the two different GBM cells at confluency either alone or in a contact co-culture with astrocytes (Figure 2A,B). As depicted in Figure 1, U-87MG and UP-007 when in a co-culture with astrocytes showed no significant difference (*p* > 0.05) in terms of rate of closure of the gap compared to a monoculture of GBM. However, it was possible to observe a positive trend in rate of closure by increasing the ratio of the astrocyte population.

### 2.3. Astrocytes Modulate Drug Sensitivity in a GBM Co-Culture Model

**Figure 3:**
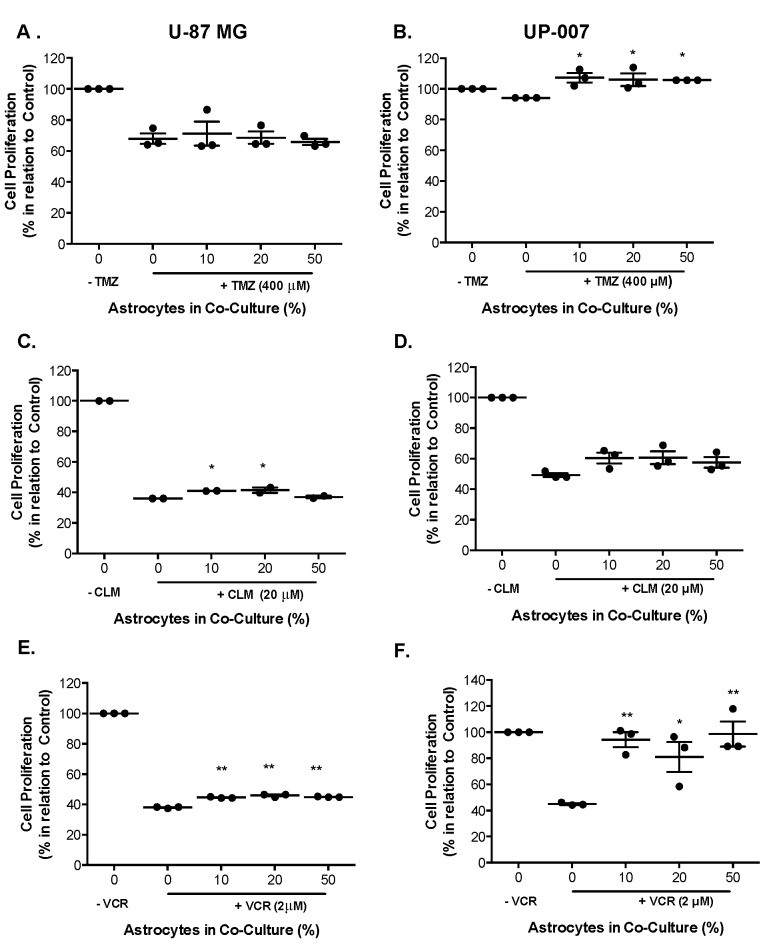
The response of GBM to temozolomide (TMZ), clomipramine (CLM), and vincristine (VCR) when in co-culture with different ratios of astrocytes in 2D. (**A**,**B**) Proliferation of U-87 MG (**A**) and UP-007 (**B**) in mono- or co-culture with UP-010 when treated with TMZ (400 μM). (**C**,**D**) U-87 MG (**C**) and UP-007 (**D**) cell proliferation in a mono- or co-culture with astrocytes after treatment with CLM (20 μM). (**E**,**F**) Proliferation of U-87MG (**E**) and UP-007 (**F**) cells in co-culture with UP-010 when treated with VCR (2 μM). Co-cultures of GBM and astrocytes were established at ratios of 90:10, 80:20, and 50:50 of GBM to UP-010. Cell proliferation is expressed in relation to control of GBM cells growing in monoculture. Mean ± SEM (*n* = 3). * *p* < 0.05, ** *p* < 0.01 in comparison to monoculture of GBM.

Astrocytes play a crucial role in GBM malignancy [44,45], however few studies have investigated their function in GBM chemo resistance to TMZ, VCR, or CLM. To delineate an effective concentration for each drug, dose-response was carried out at a range of concentrations, and the IC_50_ value was obtained (Supplementary, Table S1). To assess the influence of astrocytes on GBM drug-sensitivity we established a co-culture system of GBM and astrocytes at different ratios (90:10, 80:20, and 50:50) to mimic TME. As shown in Table S1, in all the cell lines, treatment with TMZ (200 to 1000 μM) resulted in a reduction of cell viability up to ~75–80% (Supplementary, Table S1), and with the range of concentrations tested, IC_50_ value was not obtained. However, the IC_50_ values at < 30 μM for each cell line were calculated with CLM and VCR (Table S1). Based on these results, a concentration within the IC_50_ range of CLM (20 μM) and VCR (2 μM) was used in GBM and astrocyte co-cultures. For TMZ, the concentration of 400 μM was chosen for the purpose [46]. When treated with TMZ, astrocyte cells had no statistically significant effect on the sensitivity of GBM cells, and a significant increase proliferation of UP-007 was observed with astrocytes at all ratios (Figure 3A). CLM- and VCR-treated GBM cells in co-culture with astrocytes were more responsive to cytotoxics even in the presence of a low percentage of astrocytes (10–20%) (Figure 3C–F, *p* < 0.05). These data indicate that astrocytes could be involved in human glioma cell sensitivity to TMZ, VCR, or CLM treatment, suggesting that the glial cells play fundamental roles in the tumour microenvironment with particular reference to astrocytes here in GBM drug resistance.

### 2.4. Interactions Between Astrocytes and GBM Cells

**Figure 4:**
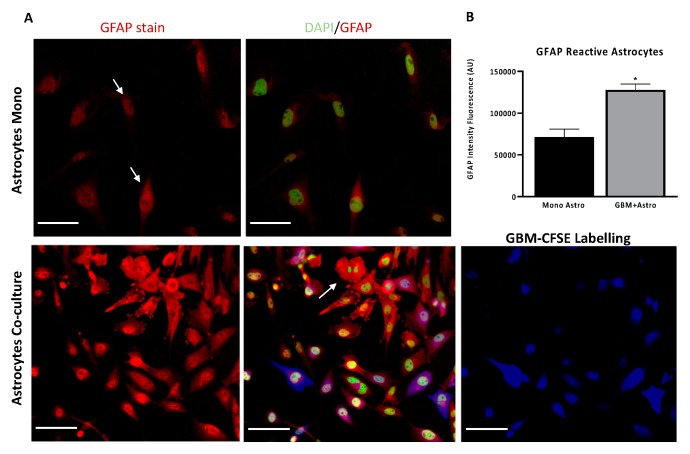
Human astrocytic cells become “reactive” in co-culture at 50:50 ratio with glioblastoma cells after 48 h. (**A**) Astrocytes co-cultured with GBM cells exhibited morphological changes and became ‘reactive’. Immunofluorescence staining for glial fibrillary acidic protein (GFAP) (Red) in normal astrocytes (UP-010) cultured alone or co-cultured with GBM (UP-007) cells. UP-007 cells were labelled with carboxyfluorescein succinimidyl ester (CFSE) (blue), and the nuclei were stained with Hoechst (in green). (**B**) Bar plot of mean fluorescence intensity of GFAP expression in astrocytes in co-culture or mono- culture. * *p* < 0.05 represents the statistical significance in a two-tailed Student’s t-test. Mean ± SEM (*n* = 2). Scale bar: 80 µm.

Previous studies have demonstrated that astrocytes interact with glioma cells triggering an astrocyte phenotypic modification by the upregulation of proteolytic enzymes and strong expression of GFAP, in particular within the tumour [47,48]. In the present study, astrocyte activation studies were conducted. An in vitro GBM and astrocyte co-culture model was established at ratio 50:50 GBM-astrocytes. As shown in Figure 4A, in the bottom panel, UP-007 GBM cells were infiltrated and surrounded by GFAP-positive activated astrocytes (red fluorescence) which exhibited morphological changes towards a multipolar star shape compared to astrocytes in monoculture which showed a classical fibroblastic form. The GFAP fluorescence intensity was also increased by ~57% (Figure 4B, *p* < 0.05) especially in co-culture where the high expression was detected in both elongated processes and perikarya of astrocytes (Figure 4A).

### 2.5. Protective Role of Astrocytes Depends on the Physical Contact with GBM 

**Figure 5:**
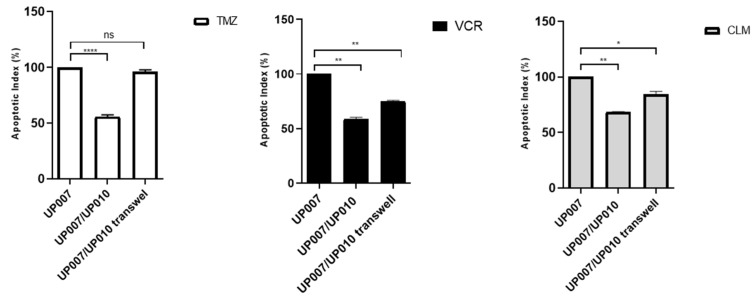
Apoptotic index of contact and non-contact cultures. The protection of GBM (UP-007) cells from apoptosis by astrocytes (UP-010) was abolished when they were physically separated by Transwell co-culture systems. The values are shown as the mean ± SEM of three experiments; not significant (ns) *p* > 0.05, * *p*< 0.05; ** *p* < 0.005; **** *p* < 0.0001.

To determine whether protection by astrocytes from therapeutic drug effect requires direct contact or paracrine signals secreted by astrocytes, we established co-culture experiments using a semi-permeable Transwell membrane, which ensures physical separation of the GBM cells from the astrocytes while allowing the transmission of secreted factors, and direct contact co-cultures. As shown in Figure 5, TMZ induced-apoptosis in GBM cells in the direct contact co-culture group was approximately 50% (UP-007/UP-010: 4.59 ± 0.13 vs. UP-010/UP-007 Tw 8.67 ± 0.89). When compared with transwell co-culture, this protection was also observed when VCR was used (UP-007/UP-010: 17.41 ± 0.007 vs. UP-010/UP-007 Transwell: 24.41 ± 2.92). When challenged with CLM the apoptosis index (Figure 5) was higher even in direct contact (UP-007/UP-010: 50.5 ± 0.28 vs. UP-010/UP-007 Transwell: 62.4 ±1.83) (Figure S3).

### 2.6. 3D Co-Culture Model Reflects the Protective Role of Astrocytes on GBM

**Figure 6:**
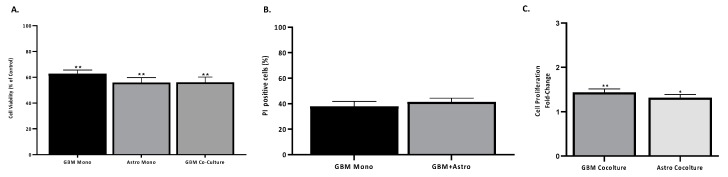
Proliferation and dead analysis on GBM and astrocytes in 3D co-culture. (**A**) Viability of mono- and co-cultures of GBM and astrocytes in the hyaluronic acid hydrogel (HyStem™-HP) compared to 2D cultures. Viability was obtained by a Cell Titer 96^®^ Cell Proliferation assay after 3 days of culture. (**B**) Propidium iodide positive cells (%) of GBM cells in mono- or co-culture with astrocytes in a 3D system was obtained by counting the number of propidium iodide (PI) negative GBM cells. (**C**) Proliferation of GBM and astrocytes in mono- or co-culture represented by CFSE-positive GBM and Far Red-positive astrocytes fluorescence. Mean ± SEM (*n* = 3). * *p*< 0.01, ** *p* < 0.05 in comparison to monoculture of GBM.

The results obtained above with the 2D co-cultures of GBM and astrocytes showed the protective effect of astrocytes on both GBM cell lines in human serum-supplemented media. Although these results demonstrate the relevance of non-neoplastic cells in in vitro models of GBM, the 3D environment is a key parameter that must be considered for pre-clinical model studies. Thus, we established a 3D in vitro co-culture mixing GBM and astrocytes using hyaluronic acid-gelatin based hydrogel aiming to mimic the extracellular matrix (ECM) of the tumour. In terms of cell viability, a Cell Titer 96^®^ Cell Proliferation assay displayed that cells, either in mono- or co-culture, growing in 3D remained viable when compared to the 2D cultures (Figure 6A). In addition, cells growing in the 3D system were stained for PI to check the number of apoptotic cells (Figure 6B). The viability of GBM cells in the 3D hydrogel was not altered by the presence of astrocytes (Figure 6B). However, in terms of cell proliferation, GBM cells in the 3D system mixed with astrocytes demonstrated a greater proliferation as the Cell Trace signal diffuses through generations (Figure 6C). Thus, these results reflect the data obtained in the 2D co-cultures, confirming that astrocytes promote proliferation of GBM. In 2D cultures, an increase in proliferation in GBM UP-007 cells was observed, while in the 3D culture in hyaluronic acid, a greater increase was obtained with growth (50:50) in co-culture, suggesting that hyaluronic acid-based ECM further supports the role of astrocytes in GBM proliferation. 

**Figure 7:**
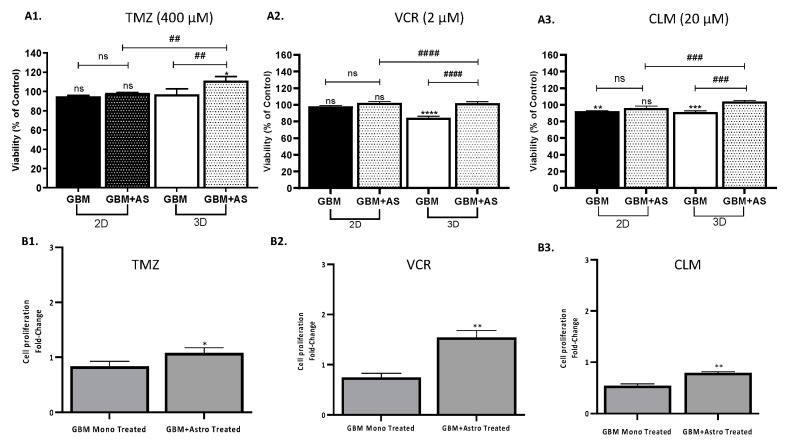
Drug response of GBM cells to TMZ, CLM, and VCR in 2D and 3D culture mono- or co-culture with astrocytes. (**A1**–**A3**) Viability of UP-007 cells when treated with TMZ (**A1**), CLM (**A2**), or VCR (**A3**) obtained by Cell Titer 96^®^ Cell Proliferation assay. Cells were cultured either in 2D or 3D conditions (HyStem™-HP) alone or in co-culture with astrocytes at ratio 50:50. Cell viability was normalized with untreated cells for each of the models used (2D/3D and mono/co-culture). Mean ± SEM (*n* = 3). * *p* < 0.05, ** *p* < 0.05, *** *p*< 0.001, **** *p* < 0.0001 compared to untreated cells. Not significant (ns) *p*> 0.05, ^##^ *p* < 0.05, ^###^ *p* < 0.001, ^####^ *p* < 0.001. (**B1**–**B3**) Corrected total cell fluorescence of GBM cultured in 3D treated with TMZ (400 µM) (**B1**), VCR (2 µM) (**B2**), or CLM (20 µM) (**B3**) either in mono- or co-culture with astrocytes. Mean fluorescence intensity (MIF) is inversely related to proliferation. Mean ± SEM (*n* = 3). * *p* < 0.05, ** *p* < 0.01 comparison to monoculture treated with the TMZ, CLM, or VCR.

Subsequently, the 3D co-culture model was tested for drug-screening using TMZ, CLM, and VCR in order to establish a comparison with the 2D co-culture model (Figure 7). Cell proliferation was assessed by Cell Titer 96^®^ Cell Proliferation assay of mono- and co-cultures of GBM and astrocytes at a ratio of 50:50 in 2D and 3D co-culture models when treated with TMZ, CLM, or VCR. In the 3D co-culture model, CLM and VCR caused a decrease of cell viability of GBM in monoculture, however a co-culture with astrocytes resulted in the survival of ~100% of the population in response to the CLM and VCR (Figure 7A2,A3). Moreover, GBM and astrocytes co-cultured in the hydrogel exhibited a 1.1- and 1.2-fold increase in cell viability compared to the monoculture of GBM in 3D when treated with CLM and VCR, respectively (*p* < 0.001). The addition of TMZ in a 3D system resulted in a statistically significant difference in the mono- and co-cultures (Figure 7A1, *p* > 0.05), however a slight increase in viability was observed when comparing both conditions in 2D and 3D. To further understand the response of GBM, cell fluorescence intensity of CFSE-positive UP-007 cells treated with TMZ, CLM, and VCR was quantified using intensity fluorescence decrease as a measure of cell proliferation (Figure 7B1–B3). In terms of cell proliferation, the dose of TMZ, CLM, or VCR did not have a statistically significant effect on the mono culture of UP-007 in 3D (*p* > 0.05). Nevertheless, when comparing the treated mono- and co-cultures in 3D, a significant reduction of the cell fluorescence was observed with all the cytotoxics indicating an increase in cell proliferation, confirming the supportive role of astrocytes to GBM sensitivity.

### 2.7. Both Homo-Cellular and Hetero-Cellular Extension Occurs Between “Reactive” Astrocytes and GBM Cells

**Figure 8:**
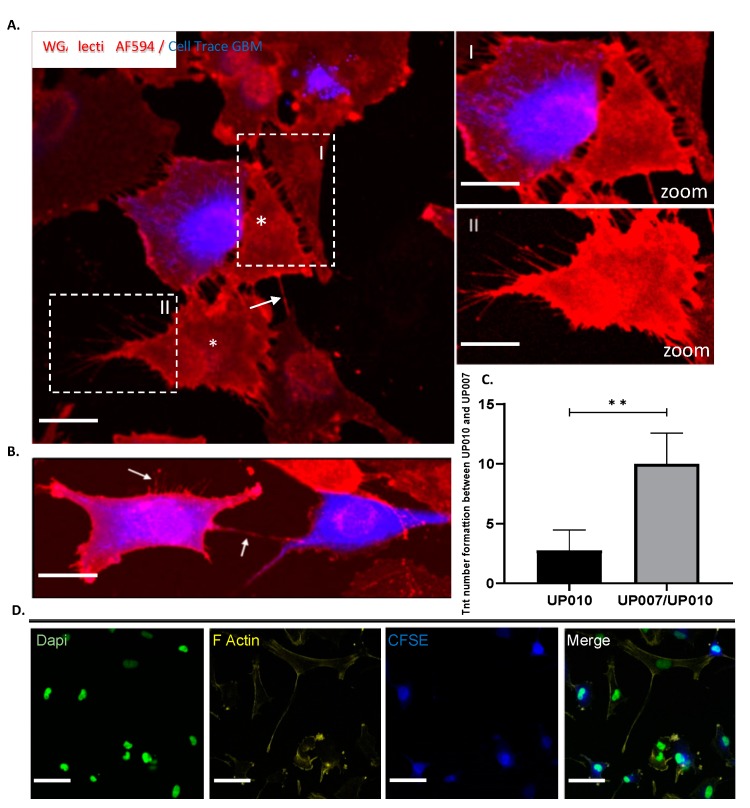
Hetero-cellular tunneling nanotubes (TNTs) connect astrocytes to GBM cells in co-culture: structural characterization. (**A**) Confocal imaging of TNTs in a co-culture system. TNTs (arrows) were formed between GBM-labelled (CFSE, blue) and unlabeled cells astrocytes (asterisks). Astrocyte (UP-010) cell lines were co-cultured with Cell Trace CFSE (blue) GBM cell lines (UP-007) for 48 h. Before imaging by confocal microscopy, the co-cultures were stained with Alexa Fluor 594 conjugated-wheat germ agglutinin (WGA AF594) to reveal cell membranes (lectins). Hetero-cellular TNTs can be observed between CFSE-positive GBM cells and astrocytes (asterisks). (**B**) GBM–GBM connections. The arrows show the established connections and the new connections around GBM cells (blue). Scale bar: 80 µm. (**C**) Numbers of TNT formations between UP-010 and UP-007 either in mono or co-culture. (** *p* < 0.01; *n* = 4).) (**D**) After co-culture, hetero cellular-type TNTs contained F-actin and microtubules. F-actin was stained with Phalloidin-AF549 (yellow). Scale bar: 50 µm.

**Figure 9:**
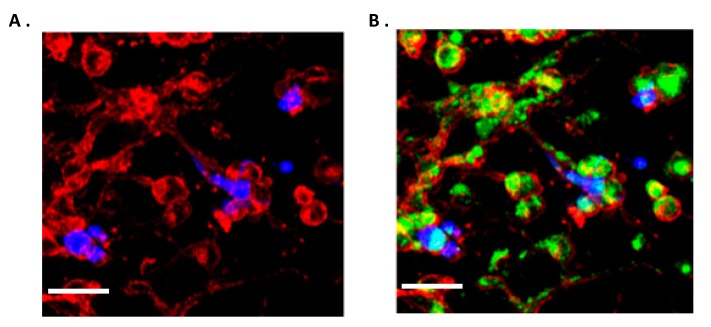
Confocal imaging shows homo-cellular and hetero-cellular extensions in 3D co-culture models using hyaluronic acid-based hydrogel and mitochondria co-localization in TNT formation. (**A**) CFSE-positive GBM (blue) cells were grown with astrocytes in HyStem™-HP hydrogel for three days and stained with WGA AF594 (red). TNTs connecting GBM cells and astrocytes. (**B**) Confocal imaging of TNTs in a co-culture system. Astrocytic cells were stained with Mito Tracker Orange FM, mixed 1/1 with CFSE GBM cells and subsequently embedded in HyStem™-HP hydrogel. Before imaging, membranes of the co-cultured cells were stained with WGA AF594. Scale bar: 50 µm.

To show the presence of this physical contact and the potential connections between astrocytes (UP-010) and GBM (UP-007) cells under co-culture conditions, we established co-cultures of 50:50 GBM and astrocytes, labelling UP-007 cells with Cell Trace fluorescent dye CFSE, and co-culturing them with UP-010 cells. Long (~10 to 200 µm) thin, tubular, membranous-based structures connecting “reactive” astrocytes (UP-010) and GBM (UP-007) as well as GBM to GBM cells were observed in 2D (Figure 8D). These processes were able to span long distances from 10 μm to 200 μm (Figure 8D) between astrocyte and GBM cells. These nano tunneling tubules between the co-cultured cells have an F-actin filaments component as shown in Figure 8D by the staining of membrane with Phalloidin WGA AF549 on confocal microscopy and that the membranous-based structures were observed after 48 h more in “reactive “astrocytes where the number of projections were increased (Figure 8C, *p* < 0.01, *n* = 4) compared to monoculture astrocytes. This suggested the TME induced the formation of these long extensions between astrocytic cells and GBM cells, enhancing intercellular communication and cytoplasmic content exchange. The nano tunneling formation and mitochondrial transfer was also observed in 3D in vitro co-culture (Figure 9).

### 2.8. TNTs Mediate Transfer of Cytoplasmic Components Between Astrocytes and GBM Cells in 2D and 3D Co-Culture Models

**Figure 10:**
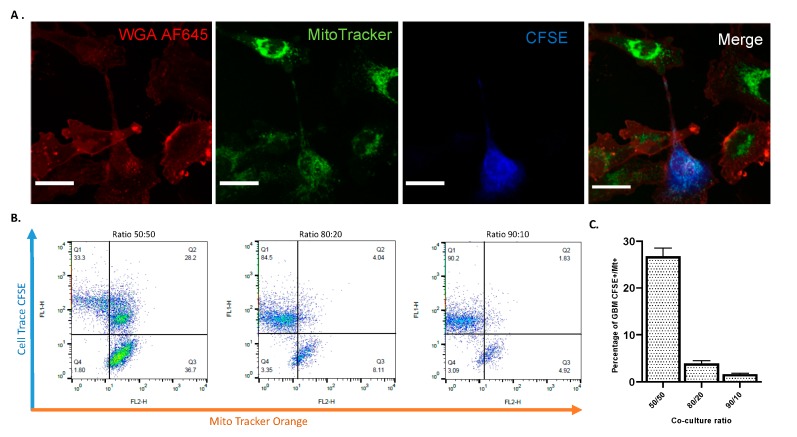
Mitochondrial transfer via nano tunneling from astrocytes to CFSE-positive GBM cells. (**A**) Confocal images of mitochondrial transfer through TNTs. Astrocytes (UP-010) were stained with Mito Tracker Orange (25 nM) to follow their mitochondria. Subsequently, they were co-cultured with GBM cells (UP-007) stained with CFSE. Prior to confocal imaging, co-cultures were stained with WGA AF594 to reveal cell membranes and TNT connections. Confocal microscopy showed Mito-Tracker Orange FM signal transferring from astrocytes to GBM cells through TNTs (white arrow) while a green dot can be seen in the recipient GBM cell (left-up panel, green arrows), and a considerable amount of Mito Tracker green signal dots could be seen in the plasma of GBM cells tracked by Cell Trace-CFSE. Scale bar: 50 µm. (**B**) Flow cytometry plot of different ratio of co-culture of GBM CFSE+ and astrocytes positive for Mito Tracker Orange. Prior to co-culture astrocytes were stained with Mito Tracker Orange FM. The number of GBM cells taking up mitochondria from astrocytes increased in relation to the number of astrocytes positive for Mito Tracker. (**C**)**.** Plot representations of the flow cytometry findings. Mean ± SEM (*n* = 3).

**Figure 11:**
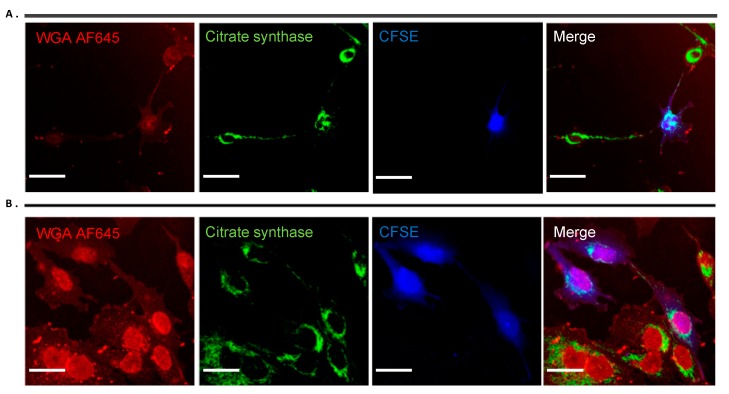
Mitochondria detection. Immunofluorescence staining in astrocytes (UP-010) and GBM (UP-007) cell lines with Anti-CS (Anti-Citrate Synthase) polyclonal antibody, showing co localization of mitochondria (green) and WGAAF645 labelling nano tunneling connections (**A**) between astrocytes and GBM cells and (**B**) between GBM cells (blue). Scale bar: 50 µm

We established both 2D and 3D co-culture experiments to investigate a possible exchange of cytosolic content through TNTs. First, GBM were labelled with CFSE to distinguish them from astrocytes in the co culture system. Prior to co culture experiments, we also labelled the astrocytes with the mitochondria-specific dye Mito Tracker Orange to observe mitochondrial transfer between astrocytes and GBM cells. After 2 days of co-culture, before quantifying the mitochondrial transfer by flow cytometry we checked nano tunneling formation and Mito Tracker staining by confocal microscopy (Figure 10A). As shown in Figure 10B,C, by increasing the astrocyte population in a co-culture with GBM, a greater mitochondrial uptake from astrocytes to GBM was obtained with the highest mitochondrial transfer at a ratio of 50:50 astrocytes to GBM (Figure 10B,C). To further corroborate these results and exclude any passive diffusion of the Mito Tracker in the culture media, we stained mitochondria using citrate synthase as a specific marker for mitochondria detection (Figure 9). Here, it is clear the movement of mitochondria along the TNTs from astrocytes to GBM as well as GBM to GBM (Figure 11A,B). The nano tunneling formation and mitochondrial transfer was also observed in 3D in vitro co culture (Figure 9A,B).

## 3. Discussion

Astrocytes have been widely reported in a variety of experimental models to modulate GBM progression and drug resistance [47,48,49,50,51,52]. However, the exact mechanism of how astrocytes facilitate GBM invasion, proliferation, and drug resistance is not yet fully understood. In order to comprehend the role of the astrocyte within the GBM microenvironment, we established a 2D co-culture model using different ratios of GBM cells to astrocytes (90:10, 80:20, and 50:50) with human serum supplementation. Establishing human and animal cells in vitro requires adequate culture conditions and culture media. In the last decade, alternative methods to avoid the use of fetal bovine serum (FBS) in cell and tissue culture media has becoming a major goal in terms of the 3R principles and in terms of good manufacturing practice (GMP), principally to guarantee safe and animal product-free conditions for biomedical tissue engineering and stem cell therapy. In our laboratories, human serum supplementation has been shown to modulate both protein and gene expression in human biopsy-derived GBM cells as well as to influence the cell shape and proliferation [53]. Human serum has also been used to expand human fibroblasts and adipose tissue-derived stem cells (ASC), showing both expansion and differentiation status of cells was superior to media supplemented with FBS [54]. To move forward with the development of pre-clinical models for drug testing studies, we established a 3D in vitro co-culture model using hyaluronic acid-gelatin based hydrogel supplemented with human serum to better simulate human in vivo conditions. 

For methods to detect different cell populations cultured together in co-culture systems, specific antigens can be employed [55,56] or genetically modified to express fluorescent proteins [57] but fluorescent dyes can also be utilized for medium/long term cell monitoring [58].

GBM is a highly heterogeneous tumour consisting of mutant cells that share many antigens such as GFAP with neural stem cells (NSCs), NSC-derived astrocytes and oligodendrocyte precursor cells (OPCs) [59] making it difficult to distinguish from astrocytic cells by the use of antibodies. In respect of this limitation and as demonstrated in our laboratory [43], without using any genetic manipulation to identify single cells, we optimized cell labelling using two amine-reactive dyes, Cell Trace CFSE and Far Red, for their flexibility, compatibility with primary cell lines and not antigen specificity. Fluorescent dye labelling was also chosen as the appropriate approach for cell tracking in order to preserve genetic heterogeneity within GBM cells. Moreover, the ability of cell trace to diffuse into daughter cells at each cell division [60], allowed us to track cell proliferation. Our results showed that CFSE (5 μM) stains the GBM cells (~99% of CFSE-positive GBM up to 7 days in culture) and is not toxic for GBM cells. Similarly, Cell Trace Far Red (1 μM) was suitable for labelling astrocytic cells maintaining them viable when compared to unstained controls. 

Human astrocytes, the most abundant cells in the glioma microenvironment, are key players in the healthy brain but also play dynamic roles in pathological conditions [61].Very few functional data on gap-junction (GJ) changes and connexin 43 (in co-culture models of rat astrocytes and GBM cells) have been reported concerning the putative role of astrocytes in supporting GBM proliferation and invasion [61,62]. Although astrocytes promote tumour progression, their influence in GBM drug response has not been investigated using all human conditions in in vitro models, particularly under human serum supplementation. We initially explored the influence of astrocytes on GBM cell proliferation and invasion using 2D direct contact co-cultures. We then translated this in a 3D in vitro model using hyaluronic acid-gelatin based hydrogels. As shown above increasing of astrocytes proliferation does not affect either GBM viability or proliferation of GBM. Zhang et al. have analyzed tumor-associated astrocytes in GBM patients and discovered similarities to astrocytes from fetal brains marked by increased proliferation [63]. Taken together, these data confirm previous results about the supportive role of astrocytes in GBM aggressiveness [9,64]. 

To comprehend the impact that astrocytes may have on GBM drug response we challenged the co-culture using TMZ, CLM, and VCR. TMZ, known as the “gold” standard option with which to treat GBM patients, acts by adding methyl groups to purine bases of DNA causing DNA damage [1], and VCR, a vinca-alkaloid that that disrupts microtubules, thus inhibiting mitosis [65], has been extensively used to treat many cancers including brain tumors [66].

CLM is a tricyclic antidepressant drug that has been shown to have selective cytotoxicity against glioma cells inducing mitochondrially mediated apoptosis [67,68,69] and autophagy [70,71]. CLM was included as, not only has it been reported to act on the GBM cells through a completely different, mitochondrial, mechanism [71,72] but because a sophisticated 3D human in vitro drug testing model may provide a non-live animal pre-clinical testing means to ‘fast-track’ re-purposed drugs like this to clinic for GBM treatment. Indeed, CLM has already shown some evidence of good tolerance and efficacy in small/anecdotal patient cohorts with 80.8% of patients from a cohort of 27 showing good partial response both clinically and radiologically [73,74,75]. Thus, the three selected cytotoxic agents act through distinct mechanisms allowing us to understand the role of astrocytes in the response of GBM to the cellular mechanisms initiated by these agents.

Based on the results above, when treated with cytotoxic agents (TMZ, CLM, and VCR), GBM cells showed an increase in proliferation when challenged with TMZ and VCR even in a low ratio of astrocytes and a slight reduction in proliferation when treated with CLM. 

Little is known about the role of tumour-associated astrocytes and their function in drug response in brain tumors. Only a handful of studies have revealed that astrocytes induce drug resistance and reduce the radio sensitivity of GBM stem cells [52]. Yang et al. using human eGFP/luciferase tagged GBM cell lines and immortalized astrocytes proposed a cell-specific bioluminescent assay for screening clinical relevant drugs [52]. Following these studies, Chen et al. demonstrated that the presence of astrocytes mediates a significantly higher cell survival after TMZ treatment in U251, C6, and A172 GBM cell lines and doxorubicin treatment in certain cell lines (U251 and LN-18) [64]. Later, using a co-culture of human astrocytes and GBM cells they confirmed the protective mechanisms of astrocytes including a reduction of glioma cell apoptosis induced by TMZ and VCR which was essentially mediated by the distribution of Ca^2+^ through Connexin 43 gap junctions [23]. Although different co-culture models have been proposed to study the interactions between astrocytes and GBM cells [47,48,49,50,51,52] most of them used rat astrocytes or selected clones of GBM fluorescent-tagged cells without considering genetic heterogeneity of a GBM cell populations. Here we employed cell labelling using fluorescence ammine CFSE and Far Red as to distinguish the two different cells population in co-culture without selecting a single tagged clone and this provided a suitable method to assess the cell proliferation without using metabolic assay test in a 3D in vitro model.

Cell-to-cell communication and TNTs have recently been discovered in the context of exchange of cytoplasmic content via extracellular vesicles (EVs) and organelles between cells, which alter biological functions [9,31,34,76,77,78,79,80,81].

Emerging evidence points to the notions that non neoplastic cells can communicate with a variety of damaged target tumour cells via TNT structures to repair and rescue damaged cells by transferring mitochondria and promoting aggressiveness and drug resistance [82,83]. Osswald et al. reported that astrocytoma cells extend ultra-long membrane protrusions, which they term “tumor microtubes” (TMs) as routes for brain invasion, proliferation, and interconnection over long distances [15]. Subsequently experiments have shown that 1p/19q non-co deleted patients’ gliomas are rich in long and intercellular TMs, while 1p/19q co deleted ones are not and pointed to a potential role for tumour microtubes in the resistance to therapies [16]. Others have demonstrated that the formation of TNTs occurs in response to micro environmental changes in ovarian cancer and other forms of invasive refractory cancers [82,83,84]. 

Here, we hypothesized from our data that physical contact between astrocytes and GBM cells and TNT formation are crucial in mediation of chemo protection and the latter provides a means to transfer cellular contents or energy between cells when are under stress. As shown in co-culture experiments “reactive” astrocytes surrounding GBM cells develop and increase TNT formation toward glioblastoma cells and these cells may use TNTs to transfer undamaged mitochondria, carrying useful substance or energy to GBM cells when challenged with cytotoxic agents. Reactive astrocytes are commonly present in brain injury [85,86] and surrounding brain tumors [87]. In stressed conditions and in response to external stimuli they typically show morphological changes moving towards a hypertrophic phenotype as well as the upregulation of the cytoskeletal intermediate filament protein GFAP [18,45,47,48]. It has also been reported that stressed conditions induce TNT formation in hippocampal rat astrocytes and neurons when treated with H_2_O_2_ or by serum depletion [88] and might facilitate cytoplasmic content transfer and therefore alter the proliferation potential of glioma cells [9]. Therefore, our data support the significance of astrocytes in the TME and the role of TNTs formation in GBM proliferation and drug resistance.

Understanding the molecular mechanisms of drug resistance in refractory cancer such as GBM is more crucial than ever in order to achieve effective cancer therapy. Drug resistance mechanisms include drug transporters, DNA damage repair (DDR) and genomic instability, inhibition of apoptosis and metabolic adaptation [89]. 

Mitochondria play fundamental roles in cancer cells, as they constitute a center for several molecular mechanisms such as apoptosis and metabolic reprogramming [89]. It has been demonstrated that tumor cells, compared to normal cells, display numerous mitochondrial dysfunctions related to energy metabolism, transmembrane potential increase, and elevated ROS generation [89,90]. Moreover, it has been demonstrated that mt-DNA is an important target of therapeutic drugs that interact with DNA [91,92].

Using fluorescent probes to track mitochondria, we observed that extensive mitochondrial transfer occurred from astrocytes to GBM cells and occurred through TNT-like structures (Figure 10, A). This data was confirmed by flow cytometry experiments showing that where there was an increased astrocyte population after 48 h in co-culture growth there was more mitochondrial exchange between astrocytes and GBM (Figure 10B,C). Most interestingly in 3D microenvironment, the ECM component acted as a supportive substrate to increase the number of TNTs (Figure 9A) as already shown in previous experiments where collagen-based matrix enhanced cell migration and process formation [93]. The presence of these structures was also confirmed by the co-localization of mitochondria within the F-actin based TNTs under both 2D (Figure 11A,B) and 3D conditions (Figure 9B). This evidence could explain the role of astrocytes within the GBM microenvironment specifically in drug response. 

Moreover, tumour-associated glial cells (TAGs) have been shown to promote GBM growth in a xenograft model [94]. Herein, we showed that glial cells, in particular astrocytes in a stressed tumour microenvironment, within both 2D and 3D in vitro co-culture models could form TNTs with GBM cells, thus reducing GBM-induced apoptosis and promoting cell proliferation and migration capacities of GBM via TNT mediated mitochondrial transfer. According to previous reports, TNTs can also transfer cytoplasmic material and organelles between two distant cells, and our study indicated that the “rescue” effect of astrocytes on glioblastoma was, at least, partially related to mitochondrial transfer via TNT-like structures.

## 4. Materials and Methods

### 4.1. Ethics Statement 

A biopsy from a glioma patient was resected at King’s College Hospital, London, under Ethics permission LREC00-173/11/SC/0048 (11 April 2011) in accordance with a National Research Ethics Service and the study was approved by ethics committees for the University of Portsmouth and King’s College Hospital (UP-007). The astrocyte cell line (UP-010) was obtained similarly from an epilepsy tissue sample under the same Ethics Permission. 

### 4.2. Cell Culture 

Two human GBM cell lines were used in this study (U-87 MG, UP-007). U-87 MG cells were obtained from European Collection of Cell Culture, UP-007 cells were established in house from the GBM biopsy resected at King’s College Hospital. UP-010 human astrocytes cell lines were established from an epilepsy tissue biopsy. GBM and astrocytes were cultured in Dulbecco’s modified Eagle’s medium (DMEM, Gibco, Fisher Scientific, Loughborough, UK) supplemented with 10% (*v*/*v*) of human serum (Sigma-Aldrich, Dorset, UK). All cells were maintained at 37 °C in a humidified incubator with 5% CO2. Media was changed every 2 to 3 days, and sub culturing was carried out by incubating cells with TrypLE Express Enzyme (Gibco, Fisher Scientific, Loughborough, UK) for 3 min. 

### 4.3. Cell Labelling

Cell labelling optimization screening was performed with all GBM cells and astrocytes using the CellTrace carboxyfluorescein succinimidyl ester (CFSE) and a Cell Trace Far Red (Invitrogen, Fisher Scientific, Loughborough, UK), respectively. For the labelling, cells were incubated with TrypLE Express for 3 min, centrifuged, and resuspended in 1 mL of HBSS (with Ca^2+^ and Mg^2+^). GBM cells were incubated with Cell Trace CFSE (5–10 μM) and UP-010 cells incubated with Cell Trace Far Red (1 μM) in HBSS for 20 min at 37 °C. Subsequently, to remove the unconjugated Cell Trace, cell suspensions were incubated with DMEM supplemented with 5% (*v*/*v*) human serum for 5 min at room temperature. Cells were centrifuged, resuspended in complete DMEM media, and analyzed in a Counter II FL Automated Cell Counter to assess the number of cells and viability. 

#### 4.3.1. Cell Labelling Efficacy

To evaluate the efficacy of the labelling, CFSE^+^ GBM cells and Far Red+ UP-010 cells were seeded at 20,000 cells/cm^2^ in 24-well plates in DMEM supplemented with 10% (*v*/*v*) human serum and allowed to grow for up to 9 days. On days 1, 3, 5, 7, and 9, cells were analyzed by flow cytometry to quantify the remaining percentage of labelled cells. CFSE-positive GBM cells (U-87 MG, UP-007) were detected using the blue 488 laser and the 530/30 channel (FL1). Far Red-positive UP-010 cells were excited with the red laser 635 and detected with a 661/16 channel (FL4). Non-stained cells were included as a control. Cells were analyzed in FACS Calibur flow cytometer (BD Biosciences, Wokingham, UK) collecting at least 10,000 events. FlowJo software (BD Biosciences, Wokingham, UK) was used for analysis of the data.

#### 4.3.2. Cell Viability

The effect of the Cell Trace on the metabolic activity of GBM cells (CFSE) and UP-010 (Far Red) was evaluated by the Cell Titer 96^®^ Cell Proliferation Assay (MTS, Promega, Southampton, UK). CFSE-positive GBM (U-87 MG, UP-007) or Far Red-positive UP-010 cells were seeded at 20,000 cells/cm^2^ in a 96-well plate in DMEM supplemented with 10% (*v*/*v*) human serum and allowed to grow up to 9 days. At specific time-points, the MTS solution was added (20 μL) to each well, and the cells were incubated at 37 °C for 2 h. Absorbance was measured at λ 492 nm using a spectrophotometric microplate reader (POLARstar Omega, BMG Labtech, Ortenberg, Germany). Non-stained cells were used as a control in each experiment. 

### 4.4. 2D Co-Cultures: Cell Viability and Proliferation 

Co-cultures of GBM (U-87 MG, UP-007) and astrocytes (UP-010) were established by seeding both cells in a 24-well plate at a final density of 20,000 cells/cm^2^. CFSE-positive GBM and Far Red+ UP-010 cells were cultured either alone (40,000 cells) or cultured in co-culture at the following ratio of GBM to UP-010 cells: 90:10 (36,000:4000), 80:20 (32,000:8000), or 50:50 (20,000:20,000). Cells were allowed to grow in close contact for 3 days. 

#### 4.4.1. Cell Viability

To evaluate the viability of the cells in co-culture (UP-007 and UP-010), cells were incubated with TrypLE Express Enzyme for ~3 min, centrifuged, and suspended in HBSS. Propidium iodide (Sigma-Aldrich, Dorset, UK) was added at a final concentration of 50 μg mL^−1^ and incubated for 5 min protected from the light. Cells were washed with HBSS and analysed in the FACS Calibur™ flow cytometer, as previously described. Propidium iodide positive cells were detected using the blue 488 laser and the 630/30 channel (FL3). FlowJo software (BD Biosciences, Wokingham, UK) was used for analysis of the data. 

#### 4.4.2. Cell Proliferation 

Cell proliferation was quantified by calculating the inverse of the fluorescence intensity of CFSE-positive GBMs and Far Red-positive UP-010. Both Cell Trace molecules readily diffuse into cells and bind covalently to intracellular amines within proteins resulting in stable, well-retained fluorescent staining. The fluorescence intensity of the Cell Trace diminished with an increase of cell division as the reagent is passed through generations, resulting in an inverse correlation between fluorescence intensity and proliferation. Thus, cells were analysed by flow cytometry, as previously described, and using FlowJo software fluorescence intensity for each channel (FL1 or FL4) was obtained. Proliferation was calculated in relation to a control of cells growing in monoculture according to equation below:

Proliferation (%) = 1/(A/B) ×100(1)

where A represents cells growing in a co-culture (90:10, 80:20, and 50:50 ratios of GBM to UP-010) and B is the control of Cells growing in the monoculture either GBM or UP-010. Cell proliferation is expressed in function of the cells growing in a monoculture.

#### 4.4.3. Cell Migration

Cell migration was evaluated using a wound-healing assay. Cells, either in a mono- or co-culture, were seeded at 100,000 cells/cm^2^ in a 24-well plate and allowed to grow to a confluent monolayer over 24 h. Afterward, a linear scratch was done using a pipette tip. Following injury, wound closure was observed using EVOS FL Auto 2 Cell Imaging System (Fisher Scientific, Loughborough, UK) equipped with a cell imaging chamber under a humidified atmosphere (37 °C, 5% CO_2_). Images were acquired every 30 min over a period of 24 h. Image analysis was performed using *Image J* (U.S. NIH, Bethesda, USA), and rate of closure (μm/h) was calculated by plotting distance of the wound gap *versus* time at different time points (0, 4, 8, 12, and 18 h).

### 4.5. 2D Co-Cultures: Drug Response

GBM (U-87 MG, UP-007) or astrocytes (UP-010) were seeded at 20,000 cells/cm^2^ in a 96-well plate and allowed to grow for 24 h. Subsequently, cells were treated with a cytotoxic: temozolomide (TMZ, Sigma-Aldrich, Dorset, UK), clomipramine (CLM, Sigma-Aldrich, Dorset, UK), or vincristine (VCR, Tocris, Bristol, UK) to calculate an IC_50_ value for each cell line. TMZ was dissolved in dimethyl sulfoxide at 1 mM, and working solutions were prepared from 200 to 1000 μM in DMEM. CLM was dissolved at 50 mM in water, and solutions were prepared at concentrations ranging 20–100 μM in DMEM. VCR sulphate was dissolved in water at 1 mM and working solutions were used from 2 to 10 μM in DMEM. After drug treatment, cells were incubated for 2 days, and Cell Titer 96^®^ Cell Proliferation Assay, as previously described, measured viability. IC_50_ values were calculated using Graph Pad Prism 7.03 software (Graph Pad, San Diego, USA). To assess the effect of astrocytes in GBM drug response, co-cultures in 2D were established as previously described, and cells were treated with TMZ (400 μM), CLM (20 μM), or VCR (2 μM). Following 48 h of drug treatment, cells were analyzed by flow cytometry using a FACS Calibur™ collecting 10,000 events. The FlowJo software was used for analysis of the data, and cell proliferation was calculated using Equation 1. Untreated cells were used as a negative control and unlabeled cells were used as a control for the flow cytometry analysis. 

### 4.6. Immunofluorescence Assay

UP-010 astrocytes and UP-007 CFSE+ GBM cells were co-cultured (50:50) in 24-well culture plates for 48 h. Cells were fixed in 4% (w/v) paraformaldehyde permeabilized with 0.5% (w/v) Triton X-100 and blocked with 1% (w/v) non-fat milk powder. Then, the cultures were incubated with primary antibodies against GFAP (Dako, rabbit monoclonal, 1:250) overnight at 4 °C, and a secondary antibody conjugated with Alexa Fluo-647 fluorescent dyes (1:1000, goat anti-rabbit IgG, Abbkine) was applied for 1 h at 37 °C. After staining with DAPI, the cells were imaged using Imaging was carried out using a LSM719 confocal laser-scanning microscope (Zeiss, Oberkochen, Germany). Mean fluorescence intensity was calculated using *Image J* Software (BD Biosciences, Wokingham, UK). 

### 4.7. Co-Culture Chemo-Sensitivity Experiments

To determine whether astrocytes could protect glioma cells from apoptosis induced by chemotherapeutic drugs, we used an astrocyte–GBM co-culture system in vitro. UP-007 cells were stained with CFSE to be distinguished from the UP-010. GBM and UP-010 astrocyte (50:50) were cultured alone or co-cultured in 6-well plates. After incubating for 24 h, the cultures were treated with TMZ (400 μM), VCR (2 µM) or CLM (20 µM). 48 h later, the cells were harvested and stained with Alexa Fluor Annexin V-647 (Invitrogen) and propidium iodide (PI) to determine the level of apoptosis of CFSE^+^ GBM cells by flow cytometry. To detect whether such sensitivity to chemotherapeutic drugs is due to direct contact between astrocytes and GBM cells or paracrine signals via astrocytes, we used a transwell co-culture system. UP-010 were seeded on top of transwell chamber with 0.4 µm-pore-sized membrane and co-cultured with UP-007 cells plated at the bottom of a 6-well plate. After adding chemotherapeutic drugs for 48 h, the apoptotic fraction was determined by flow cytometry as described below.

#### Apoptosis Determined by Flow Cytometry

The culture medium containing floating cells and the attached cells was collected from each well. Apoptosis was analyzed by flow cytometry according to the manufacturer’s instructions. Apoptotic UP-007 cells were defined as Annexin V-647-positive cells. The Annexin V-647-positive and PI-negative area represents early apoptosis, Annexin V/PI-positive area represents end-stage apoptosis and death. In co-culture systems, UP-007 cell apoptosis was detected by gating on CFSE^+^ events to exclude UP-010. Apoptotic index was calculated according to Prieto et al. [95].

### 4.8. Immunofluorescence Detection and Confocal Imaging of TNTs and Mitochondrial Transfer

#### 4.8.1. Membrane Staining and TNT Formation Processes

Cells were labelled with 1 mg/mL Alexa Fluor^®^ 594-conjugated wheat germ agglutinin (WGA, Invitrogen) at 5 μg/mL for 15 min at 37 °C in the dark. WGA is a fluorescent lectins probe for detecting glycol-conjugates, which selectively binds to N-acetylglucosamine and N-acetylneuraminic acid residues of cell membranes. 

#### 4.8.2. TNT Counts

Monoculture or Co-cultures (50:50 ratio) of astrocytes and glioblastoma cells CFSE-positive were grown for 3 days and after stained with WGA AF594 to specifically mark membrane projection in both cells. TNTs extensions were counted in 10 randomly selected fields using a 20× objective and averaged.

#### 4.8.3. Cytoskeletal Staining

To determine the composition of TNTs, astrocytes and GBM cells were seeded onto 24–well glass bottom dishes 15.000 cells/cm2 at a 50:50 ratios and growth for 48 h, subsequently the co-culture system was treated with 4% (w/v) PFA. The cells were then permeabilized with 0.1% (w/v) Triton X-100 in PBS for one minute at room temperature and washed three times with PBS. 1% (w/v) non-fat milk powder was used as a blocking agent and applied for 30 min. The sample as then stained with Alexa Fluor 546 Phalloidin (Life Technologies) and DAPI (Life Technologies) for 45 min, washed three times and stored in fluorescent mounting medium (Dako North America, Carpinteria, CA, USA).

#### 4.8.4. Mitochondrial Transfer Between Astrocyte Cells and Glioblastoma Cells via TNTs

To trace intercellular exchange of mitochondria, cells were labelled separately with Mito Tracker^®^ probe. Briefly, cells were resuspended in prewarmed (37 °C) staining solution containing the MitoTracker^®^ probe (25 nM) for 30 min under appropriate growth conditions. Cells were plated in mono or co-culture according to the experiment and allowed to grow for 3 days. To avoid potential not TNT related mechanisms of Mito Tracker^®^ transfer e.g., via uptake of cell debris from dead cells containing fluorescent particles, a medium change immediately after attachment of the living cells to the culture dish was performed. Mito Tracker^®^ passively diffuses across the plasma membrane of live cells and accumulates in active mitochondria. After staining, cells were washed three times in PBS and suspended in fresh pre-warmed medium. All imaging was performed using a Zeiss LSM719 confocal laser-scanning microscope (Carl Zeiss, Oberkochen, Germany). Post-acquisition image analysis was performed with ZEN 2012 ×32 blue software (Carl Zeiss). Images were taken with an oil immersion 40×/1.4NA objective (Carl Zeiss).

#### 4.8.5. Analysis of Mitochondrial Transfer by Flow Cytometry

To visualize Mito Tracker transfer via TNTs, we seeded GBM CFSE^+^ cells at different ratios (90:10, 80:20, and 50:50) with astrocytes stained with Mito Tracker Orange 25 nM (Invitrogen). Cells were allowed 48h to grow prior to analyses. After co-culture, cells were trypsinised, resuspended, and washed with PBS. Fluorescence (FL) was quantified on a FACS Calibour (BD Biosciences). Data were processed with FlowJo Software (BD Biosciences, Wokingham, UK). Glioblastoma CFSE fluorescence cells was acquired with (FL-1 channel) 488 nm blue laser and 510/50 nm emission, while Mito Tracker Orange astrocytes was acquired with a FL-2 Channel 640 nm red laser and 670/14 nm emission. Dot plot graph display co cultured cells detected with different channels.

#### 4.8.6. Citrate Synthase Immunofluorescence

To detect the mitochondria along Nano tunneling structures, astrocytes (UP-010) and CFSE-positive glioblastoma (UP-007) cells were co-cultured at ratio 50:50 in 24-well culture plates for 48 h. Cells were labelled with 1 mg/mL Alexa Fluor^®^ 594-conjugated wheat germ agglutinin (WGA) (Invitrogen, Fisher Scientific, Loughborough, UK)) at 5 μg/mL for 15 min at 37 °C in the dark and permeabilized with 0.2% (w/v) Triton X-100. The cultures were then incubated with primary antibodies against Citrate Synthase (GeneTex, rabbit polyclonal, 1:2000) overnight at 4 °C, and a secondary antibody conjugated with Alexa Fluo-488 fluorescent dyes (1:1000, goat anti-rabbit IgG, Jackson Immuno-research) was applied for 1 h at 37 °C. All imaging was performed using a Zeiss LSM719 confocal laser-scanning microscope (Carl Zeiss, Oberkochen, Germany). Post-acquisition image analysis was performed with ZEN 2012 ×32 blue software (Carl Zeiss). Images were taken with an oil immersed 40×/1.4NA objective (Carl Zeiss).

#### 4.8.7. Mitochondrial and Nano Tunneling Detection in 3D Co-Culture Model

To visualize mitochondria and Nano tunneling within 3D in vitro co-culture model cells were grown co-culture, in a 3D HyStem™-HP hyaluronic acid-based hydrogel (Sigma-Aldrich, Dorset, UK). HyStem-HP hydrogel is formed when the crosslinking agent Extralink™ (polyethylene glycol diacrylate Mw 3400 g/mol) is added to a mixture of HyStem-HP (thiol-modified hyaluronan) and Gelin-S™ (thiol-modified gelatin). Hydrogel was prepared according to the manufacturers’ instructions. GBM (UP-007) and astrocytes were grown in a co-culture at the ratio of 50:50 in the hyaluronic acid-based hydrogel. Briefly, UP-007 and UP-010 cells were labelled with Cell Trace CFSE and Mito Tracker, respectively, and encapsulated within the liquid hydrogel precursor solution at 1 × 10^6^ cells mL^−1^. Hydrogels were allowed to polymerize for 20 min, and then complete DMEM media was added to each well. Cells in 3D were allowed to grow for 3 days. Before collection of pictures, the 3D model was stained with WGA AF594, following the protocol. All imaging was performed using a Zeiss LSM719 confocal laser-scanning microscope (Carl Zeiss, Oberkochen, Germany). Post-acquisition image analysis was performed with ZEN 2012 ×32 blue software (Carl Zeiss).

### 4.9. Statistical Analysis

The results are expressed as mean ± standard error of the mean (SEM). One-Way ANOVA obtained comparisons between groups using the Dunnett’s post-hoc test in comparison to a control (GraphPad Prism 7.03, San Diego, USA). A significance level of *p* < 0.05 was considered statistically significant. In each experiment, *n* = 3 replicates were carried out for each condition and the data displayed represents the mean of three independent experiments.

## 5. Conclusions

In this paper, we highlighted the importance of having a human astrocyte component as well as the tumour cells grown in human serum-supplemented media to develop a highly sensitive and reproducible method to test relevant clinical drugs in this novel high-throughput, 3D in vitro, all human model. We also proposed the feasibility of using fluorescence amine staining CFSE and Far Red to distinguish two different cells in co-culture avoiding the use of genetic manipulation and clonal selection. This procedure allowed us to monitor cells using a sophisticated approach i.e., flow cytometry and confocal microscopy for pharmacological screening in a high throughput, 3D all human in vitro model and allowed us to explain the possible mechanism behind GBM drug resistance. Moreover, we showed a mechanism for drug resistance by way of TNTs delivering non neoplastic cell mitochondria. In particular, in the presence of non-neoplastic astrocytes within a hyaluronic acid-rich human serum-supplemented 3D model, GBM cells show an increase in proliferation when exposed to a therapeutically relevant dose of both TMZ or VCR while when treated with CLM there was a slight reduction in proliferation.

## Supplementary Materials

Supplementary Materials can be found at https://www.mdpi.com/1422-0067/20/23/6017/s1.

## Abbreviations

GBM	Glioblastoma
FBS	Fetal bovine serum
eGFP	enhanced green fluorescent protein
2D	Two dimensional
3D	Three dimensional
WGA	Wheat germ agglutinin
VCR	Vincristine
CLM	Clomipramine
TMZ	Temozolomide
TNT	Nano tunneling
TW	Transwell
CFSE	Carboxyfluorescein succinimidyl ester
ECM	Extracellular matrix component
TME	Tumour microenvironment
