# Supplementary material for: Pre-Clinical Drug Testing in 2D and 3D Human In Vitro Models of Glioblastoma Incorporating Non-Neoplastic Astrocytes: Tunneling Nano Tubules and Mitochondrial Transfer Modulates Cell Behavior and Therapeutic Respons
Source: Int J Mol Sci. 2019 Nov 29;20(23):6017. doi: 10.3390/ijms20236017 (PMC6929151; doi:10.3390/ijms20236017)
Supplement: Supplementary file 1 [file ijms-20-06017-s001.pdf]

Supplementary Data.

Table S1. IC_{50} values of TMZ, CLM and VCR in three GBM cell lines and astrocytes.

IC_{50} (µM)*	Temozolomide	Vincristine	Clomipramine
**GBM**			
U87-MG	>1000 µM	3.60 ± 0.62 µM	16.37 ± 1.53 µM
UP-007	>1000 µM	6.92 ± 1.77 µM	6.48 ± 2.20 µM
**Astrocytes**			
UP-010	>1000 µM	0.14 ± 0.48 µM	2.4 0± 0.43 µM

***IC_{50} value are mean ± standard error of means calculated from 3 independent experiments.**

Figure S1.

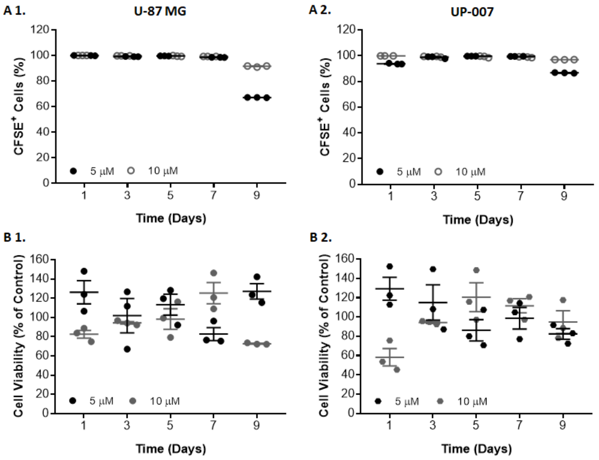

Fluorescent labelling and viability of GBM cells marked with Cell Trace. A1-A2) Percentage of CFSE-positive U-87 MG (**A1)** and UP-007 (**A2**) over 9 days of culture. Percentage of cells is given compared to an unstained control. B1-B2) Viability of U-87 MG (**B1**), UP-007 (**B2**) cells stained with Cell Trace CFSE (5-10 µM) compared to unstained control cells. Mean ± SEM (N=3).

Figure S2.

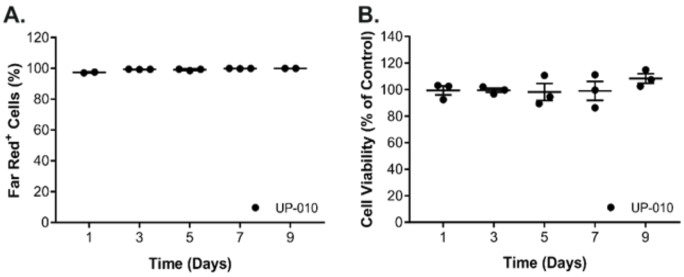

Fluorescent labelling and viability of astrocytes (UP-010) marked with Cell Trace Far Red. **A**) Percentage of Far Red-positive UP-010 cells along 9 days of culture. Percentage of cells is given compared to an unstained control. **B**) Viability of Far Red-positive UP-010 compared to unstained control cells. Mean ± SEM (N=3).

Figure S3.

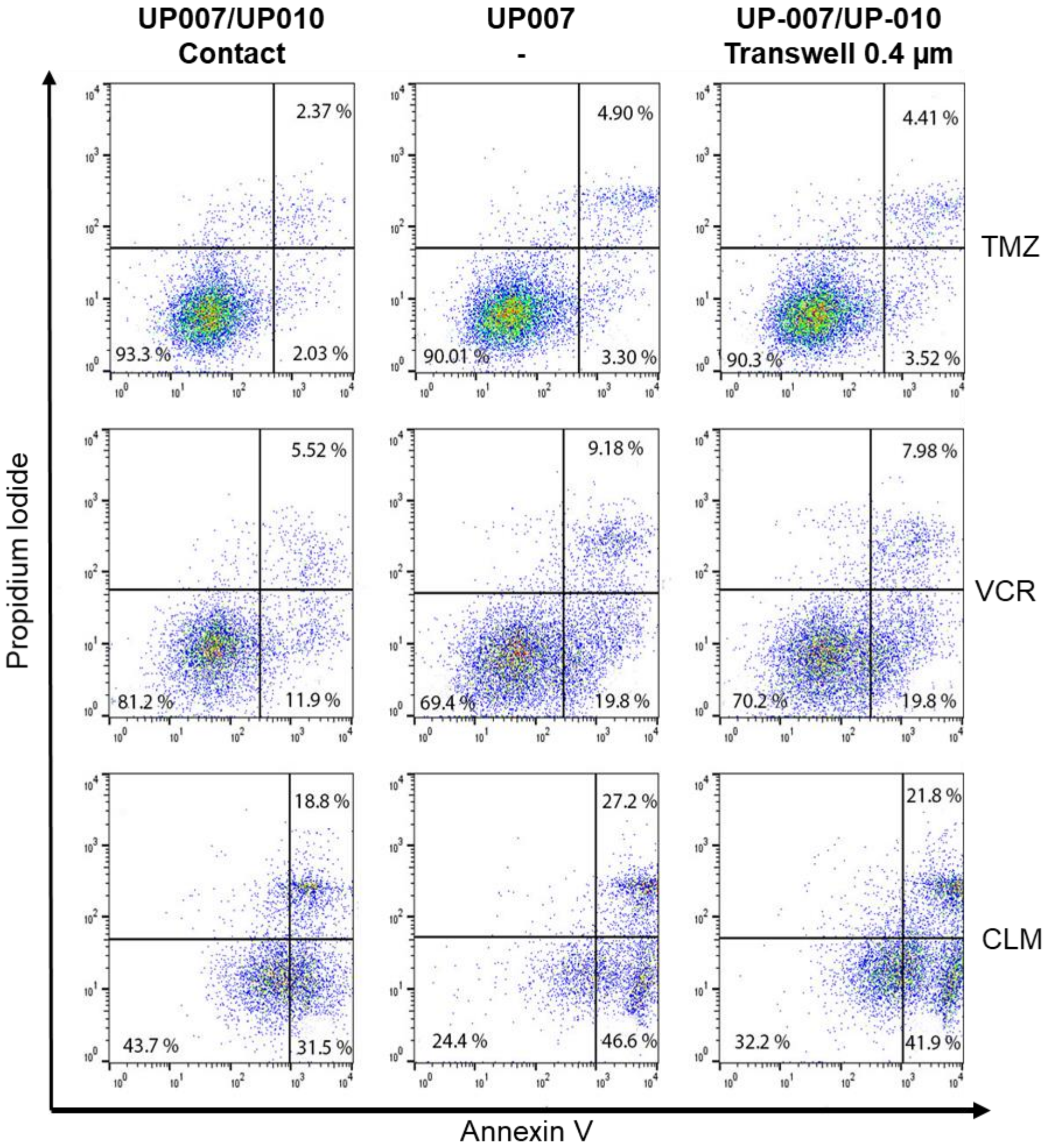

Astrocytes protect glioblastoma cells from apoptosis by direct contact. To avoid cell-to-cell contact astrocytes cells (UP-010) were seeded on the top of transwell chamber (pore size 0.4 μm) while the CFSE glioblastoma cells (UP-007) were seeded on the bottom of 24 well plate. Representative dot plot showed TMZ (400 μM), VCR (2 μM) or CLM (20 μM) induced apoptosis of UP-007 in different culture system.
